# Leopard (*Panthera pardus*) occupancy in the Chure range of Nepal

**DOI:** 10.1002/ece3.8105

**Published:** 2021-09-21

**Authors:** Babu Ram Lamichhane, Saneer Lamichhane, Rajan Regmi, Milan Dhungana, Shyam Kumar Thapa, Anil Prasai, Aashish Gurung, Santosh Bhattarai, Rajan Prasad Paudel, Naresh Subedi

**Affiliations:** ^1^ National Trust for Nature Conservation Lalitpur Nepal; ^2^ President ChureTerai Madhesh Conservation Development Board Lalitpur Nepal; ^3^ Graduate School of Veterinary Medicine Hokkaido University Sapporo Japan

**Keywords:** Chure range, Leopard, Nepal, occupancy modeling, spatial replicate model

## Abstract

Conservation of large carnivores such as leopards requires large and interconnected habitats. Despite the wide geographic range of the leopard globally, only 17% of their habitat is within protected areas. Leopards are widely distributed in Nepal, but their population status and occupancy are poorly understood. We carried out the sign‐based leopard occupancy survey across the entire Chure range (~19,000 km^2^) to understand the habitat occupancy along with the covariates affecting their occupancy. Leopard signs were obtained from in 70 out of 223 grids surveyed, with a naïve leopard occupancy of 0.31. The model‐averaged leopard occupancy was estimated to be 0.5732 (*SE* 0.0082) with a replication‐level detection probability of 0.2554 (*SE* 0.1142). The top model shows the additive effect of wild boar, ruggedness, presence of livestock, and human population density positively affecting the leopard occupancy. The detection probability of leopard was higher outside the protected areas, less in the high NDVI (normalized difference vegetation index) areas, and higher in the areas with livestock presence. The presence of wild boar was strong predictor of leopard occupancy followed by the presence of livestock, ruggedness, and human population density. Leopard occupancy was higher in west Chure (0.70 ± *SE* 0.047) having five protected areas compared with east Chure (0.46 ± *SE* 0.043) with no protected areas. Protected areas and prey species had positive influence on leopard occupancy in west Chure range. Similarly in the east Chure, the leopard occupancy increased with prey, NDVI, and terrain ruggedness. Enhanced law enforcement and mass awareness activities are necessary to reduce poaching/killing of wild ungulates and leopards in the Chure range to increase leopard occupancy. In addition, maintaining the sufficient natural prey base can contribute to minimize the livestock depredation and hence decrease the human–leopard conflict in the Chure range.

## Graphical abstract

The model‐averaged leopard occupancy in the Chure range (~19,000 km^2^) was 0.5732 (*SE* 0.0082) with a detection probability of 0.2554 (*SE* 0.1142). The top model included wild boar, ruggedness, presence of livestock, and human population density as covariates. Further, maintaining a sufficient natural prey base can contribute to minimize the livestock depredation and hence decrease the human–leopard conflict in the Chure range.

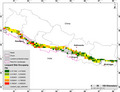

## 1 INTRODUCTION

Common leopard (*Panthera pardus,* called “leopard” hereafter) is a widely distributed large carnivore adapted to a multitude of habitats and tolerant to live in proximity of humans (Athreya et al., 2016; Hunter et al., 2013; Myers, 1986; Nowell & Jackson, 1996; Sunquist & Sunquist, 2002). Despite their high adaptability, they require a large area with abundant prey for survival, thus, threatened by landscape fragmentation, prey depletion, poaching, conflict with humans, and trophy hunting (Athreya et al., 2011; Cardillo et al., 2005; Jacobson et al., 2016; Karanth, 1999; Kissui, 2008; Raza et al., 2012; Strampelli, 2015; Walston et al., 2010). The leopard is now confined to 25%–37% of its historical range (Cardillo et al., 2005; Jacobson et al., 2016) and listed as “Vulnerable” in IUCN redlist (IUCN, 2020). Globally, only 17% of the leopard habitat lies inside the protected areas (PAs; Jacobson et al., 2016). Intact PAs play a significant role for many large carnivores, but for leopards, conservation cannot be ensured only in the PAs (Balme et al., 2010; Strampelli, 2015; Swanepoel et al., 2013; Woodroffe & Ginsberg, 1998).

The leopard habitat outside protected areas is rapidly declining, and within Pas, they face exploitative and interference competition with the socially dominant large carnivores such as tigers (*Panthera tigris*) and lions (*Panthera leo*) in most of their distribution range (Barber‐Meyer et al., 2013; McDougal, 1988; Miller et al., 2018; Miquelle et al., 2005; Seidensticker, 1976; Seidensticker et al., 1990). Among the mammalian carnivores, the less efficient competitors avoid the specialized competitors through spatial segregation by establishing the home range outside of the specialized competitors (Atwood & Gese, 2010; Grassel et al., 2015; Gubbi et al., 2020; Thapa et al., 2021; Thornton et al., 2004).

**FIGURE 1:**
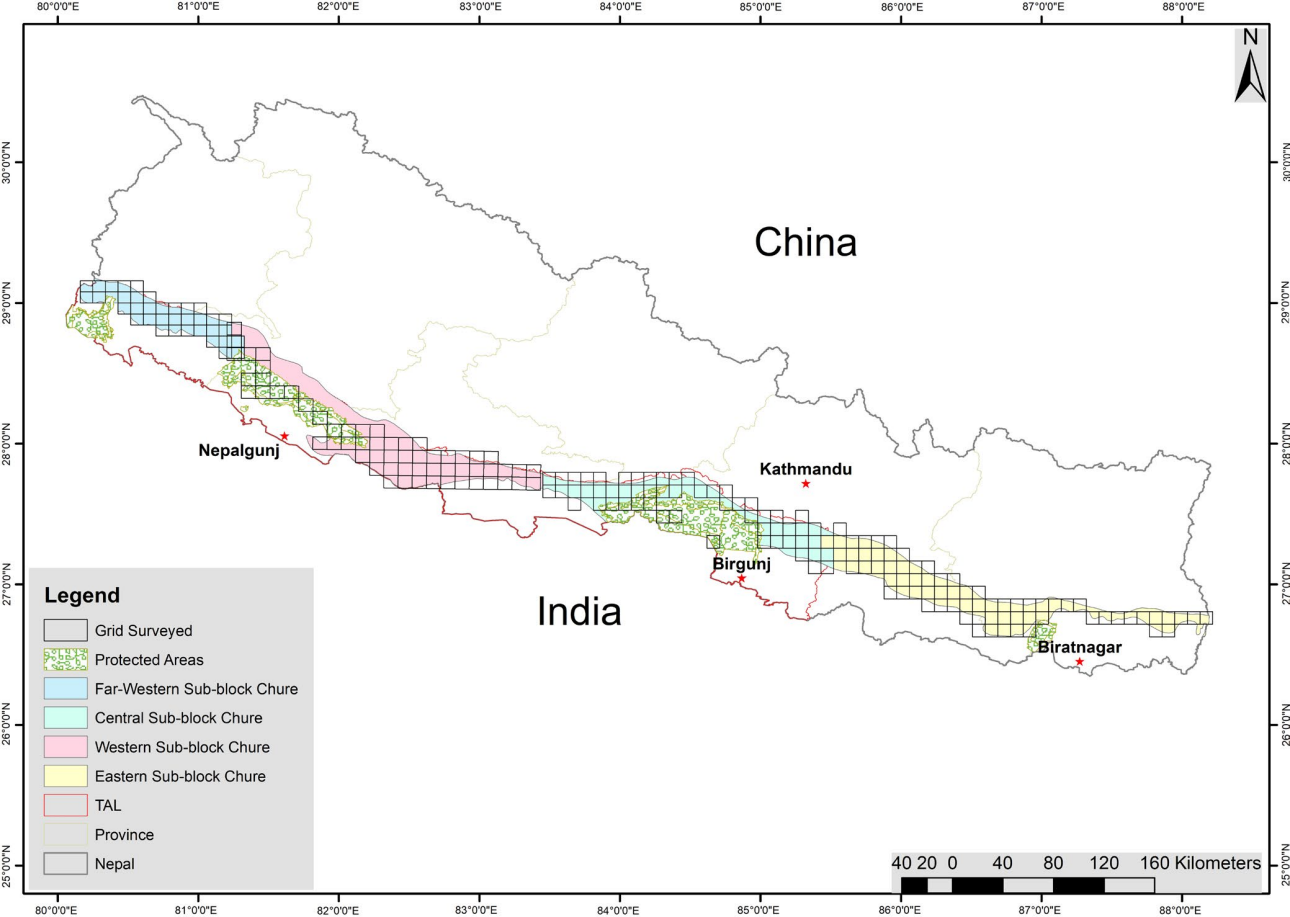
Chure range: divided into four blocks which were further divided into 10 km × 10 km grids. Each color represents each block and boundary of Chure. The blocks are in the order (from east to west): eastern block (yellow color), central block (light blue color), west block (pink color), and far‐west block (blue color). In our analysis, the Chure range was also divided into east Chure (includes eastern block) and west Chure (includes rest three blocks west from the eastern block). The lowland protected areas are shown, which is five in the west Chure and one in the east Chure. Kathmandu is the capital of Nepal, and others are the major cities in the lowland of Nepal

In Southern lowlands and Himalayan foothills of Nepal, the leopards coexist with tigers in the National Parks and Buffer Zone areas (DNPWC & DoFSC, 2018; Subedi, Bhattarai, et al., 2021; Subedi, Lamichhane, et al., 2021). Further, a recent camera trap study in the Chure range detected tigers in Kapilvastu, Palpa, and Rupandehi districts between Chitwan National Park and Banke National Parks (Subedi, Bhattarai, et al., 2021; Subedi, Lamichhane, et al., 2021). The tiger populations in Nepal have almost doubled since 2010 through tiger‐focused conservation activities in and around the tiger bearing PAs (DNPWC & DFSC, 2018; Thapa et al., 2017). Thus, the increasing number of tigers may have pushed leopards to marginal habitats with some resource overlapping (Kafley et al., 2019; Lamichhane, Leirs, et al., 2019). A large part of the Chure range falls outside the PAs. The forested areas of the Chure range adjoining the PAs provide habitat for dispersing wildlife population including the leopards (Figure 1). Tigers are primarily confined to protected areas and connected forest patches, and a large part of Chure is unoccupied by them. Thus, the Chure forest provides an opportunity for leopards to occupy a large area as an apex predator (Thapa & Kelly, 2016, 2017; Thapa et al., 2021).

Although Chure range has a potential of being key wildlife habitat for leopards and other associated wildlife, with increasing human pressure, the fragile Chure range has high deforestation rate (FRA/DFRS, 2014) which will affect in the abundance and distribution of wildlife (GoN‐RCTM, 2017). In addition, there is no comprehensive study on the status and distribution of wildlife in the Chure range. We carried out this study as a part of faunal diversity assessment in forests of Chure range (~70% of the total Chure range) of Nepal to understand the distribution and occupancy of leopards. This study provides information on leopard occupancy and associated covariates in Chure range of Nepal with far‐reaching implications for the conservation of leopards in the human‐dominated landscapes of Nepal and elsewhere.

## 2 MATERIALS AND METHODS

### 2.1 Study area

The study was carried out in the Chure range (18,982 km^2^) of Nepal. Chure is the young mountain range consisting of fragile sedimentary rocks such as mudstones, shale, sandstones, siltstones, and conglomerates (Pokhrel, 2013). It extends from east to west in southern Nepal spread in all the seven provinces (Figure 1). Chure has monsoon‐dominated subtropical climate. The average maximum and minimum temperature of this range lies between 15.8 and 31.8°C. The mean annual precipitation is between 1,400 mm and 2,000 mm (FRA/DFRS, 2014; GoN‐RCTM, 2017). The Chure range has highly rugged terrain, and the altitudinal variation ranges from 120 to ~2,000m. Over 160 river systems with a different origin flow through this range (Chaudhary & Subedi, 2019; FRA/DFRS, 2014; GoN‐RCTM, 2017).

A large part of the Chure range (>70%) is forested and is the potential habitat for various wildlife such as leopards. The range consists of 23.4% of the forests nationally and 3.5% of other woodland covers of Nepal (FRA/DFRS, 2014). This range is important for biodiversity and represents three ecoregions, nine forest types, eight important plant areas (IPAs), 14 important bird areas (IBAs), and six protected areas (FRA/DFRS, 2014). This range acts as a water reservoir for the Terai region toward the south where more than half of Nepalese people live. The government of Nepal has initiated the conservation of this range via President Chure‐Terai Madhesh Conservation Development Board. The central and western part of Chure falls in the Terai Arc Landscape (TAL) which is a globally significant the landscape for biodiversity (MoFSC, 2015). The Chure serves as an important habitat for endangered and threatened wildlife including tiger, greater one‐horned rhino (*Rhinoceros unicornis*), Asian elephant (*Elephas maximus*), leopard, gaur (*Bos gaurus*), sloth bear (*Melursus ursinus*), pangolins (*Manis crussicaudata* and *M. pentadactyla*), and hyena (*Hyaena hyaena*). Ungulates such as wild boar (*Sus scrofa*), barking deer (*Muntiacus vaginalis*), sambar (*Rusa unicolar*), chital (*Axis axis*), and three primates rhesus monkey (*Macaca mulata*), Assamese monkey (*Macaca assamensis*), and Terai gray langur (*Semnopithecus hector*) serve as prey species for a range of carnivores including the leopards.

Chure is the home for 14% of Nepal's human population, and only 14% of the Chure area is suitable for cultivation (SAWTEE, 2016). The majority of the people depend on subsistence farming for food crops, and animal husbandry is an integral part of their farm. Livestock grazing is widespread across the Chure forests. Deforestation, unplanned road construction, agricultural practices on the steep slopes, drying of the water resources, lowering of the water table, and climate change are affecting this range (Bhandari et al., 2016; Chaudhary & Subedi, 2019; FRA/DFRS, 2014; GoN‐RCTM, 2017; Pokhrel, 2013).

### 2.2 Study design

The Chure range was divided into 4 blocks (size ~2,200–6,400 km^2^) for easy organization of the survey. Each block was further divided into grids of size 10 × 10 km^2^ and surveyed in two to three shifts successively. We chose 10 × 10 km^2^ grid size because it was larger than the home range size of leopards, that is, 6–90 km^2^ in lowland Nepal and similar habitats (Norton & Henley, 1987; Odden & Wegge, 2005; Seidensticker, 1976; Simcharoen et al., 2008). We sampled the entire Chure, and thus, results reflect the true occupancy, that is, the proportion of area occupied by leopard at landscape level (Karanth et al., 2011; Thapa et al., 2021). Biologists and wildlife technicians (*n* = 12) with over 5 years of field experience in wildlife research conducted the survey in the field. The survey team was trained on survey protocols and wildlife sign identification before starting the survey to ensure the quality of the data. Out of 322 grids cells in the entire Chure range, 223 were surveyed which falls in the forested areas. The rest of the grids (*n* = 109), which either fall entirely outside of the forests or was inaccessible due to undulating steep rugged terrain, were omitted from our study. Each grid was further divided into 16 subgrids of 2.5 × 2.5 km^2^ (*n* = 3,568) for the uniformity to search the presence of leopard sign and associated covariates influencing their occupancy and detection. The survey was conducted between 2016 and 2018. We could not cover the entire Chure range in a single year due to the large area and limited human resources available. We carried out the survey in the same season (postmonsoon) to avoid the potential bias from surveys in different years.

A 2‐km‐long continuous random walking transect (defined as search paths; Thapa et al., 2021) with four segments of 500 m was surveyed within a subgrid, with maximum of 32‐km search paths within each grid; that is, the encounter occasions limit to 16 spatial replicates of 2 km each. However, these expected survey efforts within each grid differ from actual survey effort in the field due to logistical constraints (Harihar & Pandav, 2012). We targeted the existing trails and dirt roads (where possible) to minimize the likelihood of false absences. We recorded the presence/absence of the tracks, fresh droppings, and other signs (feeding sign, territory marking, etc.) to detect the presence of leopards, tigers, and large (>55 kg), medium (20–55 kg), and small‐sized prey species (<20 kg) (Lamichhane, Leirs, et al., 2019) at each segment in the standard data format as sample covariates. The leopard pugmark was differentiated from tiger from their smaller sizes such as pad size width (<6.5 cm, tiger = 9–10 cm), front foot width (~9 cm leopards, tiger = 12–14 cm), adult stride length (~90 cm, tiger = >100 cm), and claw‐scraping (<25 cm height and <15 cm width; tiger = >35 cm height and >19 cm width). Further, the tiger scat diameter is >2.5 cm (Reddy et al., 2004) and has a lower degree of coiling and a relatively larger gap between two successive constrictions (Andheria et al., 2007; Biswas & Sankar, 2002; Wang & Macdonald, 2009). The prey species were identified through their pellets, and track shape and size. The track size of rhesus (circular forehands ~6cm, elongated hind tracks ~6.5–8 cm), spotted deer (length of male = 5–6.6 cm, female = 3.5–5cm, width = 3.8–4.5 cm, elongated track), barking deer (3.5–4.9 cm length, 3–3.7 cm width, sharp edges cutting deep into the soil), wild boar (5.0–6.5 cm length, two dewclaws may mark on the soil and their anterior section marks deeply), goats (4.5–5.8 cm length, 4.8 cm width), cows (<10 cm length, outer hoof surface is well marked), and buffalo (10–12 cm length, front hoof section marks deep were referred from Menon and Daniel (2009) and Kolipaka (2014)). Similarly, the human pressure as lopping, encroachment, and livestock presence was recorded in each segment.

### 2.3 Occupancy modeling

The naïve occupancy was calculated by dividing the number of grids with species present/total number of grids surveyed in the block. We used program PRESENCE (2.12.33) to obtain the true occupancy of leopards of Chure range (MacKenzie et al., 2002). We applied the single species occupancy model with correlated replicate surveys, which explicitly take into account the spatial correlation in detection across the 2‐km continuous random walking transect (search paths) within each grid. It is because the leopards can travel greater than the size of our replicate (2 km) per day; hence, the detection of the sign in successive spatial replicates violates the statistical independence required by the standard occupancy model (MacKenzie et al., 2017). The spatial correlation model (Hines et al., 2010) accounts for this correlation in the detection using the Markov spatial dependence approach. For the degree of dependence between the replicated samples, the model uses replicate‐level occupancy parameters “*θ*_0_” and *θ*_1_, where “*θ*_0_” = Pr (leopard presence in a replicate/grid occupied and which was absent in the previous replicate) and “*θ*_1_” = Pr (leopard presence in a replicate/grid occupied and was present in the previous replicate). We also checked the performance of the standard occupancy model (MacKenzie et al., 2002) and spatial correlation model (Hines et al., 2010) without adding any covariates in our data. We compared these models based on the Akaike information criterion (AIC) and chose one with lowest AIC scores (Burnham & Anderson, 2002). It clearly showed the spatial dependencies in sign detection on 2‐km long replicates with less AIC value (better performance) for the spatial correlation model compared with the standard occupancy model (Table 1). Hence, all other analyses were performed using spatial correlation model (Hines et al., 2010).

**TABLE 1:** Model selection between spatial correlation and standard occupancy model

Model	AIC	ΔAIC	*w*	Model likelihood	*K*
*Chure range*					
$\hat{\Psi }$ *θ*_0_(·) *θ*_1_(·) *p*()*θ*_0_ *pi*(·)	939.41	0	1	1	20
$\hat{\Psi }$(·),*p*(·)	990.25	50.84	0	0	17
*East Chure*					
$\hat{\Psi }$(·),*p*(·)	262.17	0	0.9523	1	17
$\hat{\Psi }$ *θ*_0_(·) *θ*_1_(·) *p*(), *θ*_0_ *pi*(·)	268.16	5.99	0.0477	0.05	20
*West Chure*					
$\hat{\Psi }$ *θ*_0_(·) *θ*_1_(·) *p*(), *θ*_0_ *pi*(·)	676.43	0	1	1	20
$\hat{\Psi }$(·),*p*(·)	736.91	60.48	0	0	17

Note

$\hat{\Psi }$: model‐averaged leopard occupancy; *p* = replicate‐level detectability; AIC = Akaike's information criterion, ΔAIC = difference in AIC value between the top model and the focal model; *w* = AIC weight; Model likelihood is −2 logarithm of the likelihood. *θ*_0_ = Pr (leopard presence in a replicate/grid occupied and which was absent in the previous replicate) and “*θ*_1_”= Pr (leopard presence in a replicate/grid occupied and was present in the previous replicate); *k* = number of model parameters; (·) = parameters are held constant. The models with lowest AIC values were chosen.

Next, we identified suitable sample and site covariates that could potentially explain any heterogeneity in leopard occupancy. For this, we predicted the effect of covariates in detectability and occupancy of leopards. We priori expected that the prey abundance and human disturbance (lopping and human encroachment) across the grid influence the leopard occupancy positively and negatively, respectively (Harihar & Pandav, 2012; Jhala et al., 2010; Karanth et al., 2011). Further, we expected the livestock negatively influences the leopard occupancy as it can be considered as a substitute for human impact (Karanth et al., 2011). Similarly, the increased human population density across the grid raises the human disturbance and hence has negative influence on leopard occupancy. Likewise, we expected the positive influence of management regime on leopard occupancy and detection as the survey grid inside the protected areas has lower disturbance compared with outside. Also, we predicted normalized difference vegetative index (site covariate, NDVI) positively influences the occupancy by providing cover and increasing opportunity for leopard, an ambush hunter, to hunt (Sharma et al., 2015), and negatively influence the detection (thick vegetation and leaf litter reduce the chances of sign detection or direct observation of leopard on the search path). Similarly, the terrain ruggedness positively influences both the occupancy and the detection of leopards as increased ruggedness will be harder for people to access and hence lowers the disturbance (Johnson et al., 2020). We also expected sampling effort (total km of search path in a grid) positively influences the detection of leopards as it may vary between the survey grids due to logistic constraint (Harihar & Pandav, 2012). We prepared a list of nine a priori hypotheses (Appendix 6).

The sample covariates collected from the field survey included prey species, PS = (barking deer, wild boar, chital, and rhesus), human disturbance (HD = lopping, human encroachment), and livestock presence (*L*). We separated the wild boar (*W*) from other prey species because many studies reported leopards avoiding the wild boar (Karanth & Sunquist, 1995; Ramakrishnan et al., 1999), and we wanted to know how wild boar affects the presence of a leopard. Moreover, the occurrence of wild boar was the most widespread among the prey species.

The site covariates were management regime (IO = inside or outside of the national park), vegetation cover measured as NDVI—Normalized Difference Vegetation Index (N), terrain ruggedness index (*R*), and human population density (PD). If a grid falls more than half inside the national park or buffer zone, it was coded as “1” and “0” if it falls outside. The human population density (PD) was obtained from the Gridded Population of the World Version 4 (GPWv4; CIESIN, 2018), and NDVI was obtained from the 250‐m resolution Medium Resolution Imaging Spectroradiometer (MODIS) satellite images of 2019 (Didan et al., 2015) available at https://earthexplorer.usgs.gov. Similarly, the terrain ruggedness index (*R*) for each grid was calculated using 90 m ASTER DEM (Fujisada et al., 2005). We averaged them across the grid surveyed using the *z*‐statistic in ArcGIS 10.1. We also included sampling effort (Samp_Eff) as a covariate that affects the detection probability. Before adding the covariates in our analysis, we tested the Spearman correlation coefficient (*r*) using PAST version (4.0) (Hammer et al., 2001) and one was dropped when a set of two covariates have |*r*| ≥ .7. Among the covariates we used, human disturbance (lopping and encroachment) and livestock were highly correlated (Appendix 7) and we used livestock to obtain the final model (Kandel et al., 2020; Kshettry et al., 2018; Reynaert, 2018). The data were prepared in an excel sheet via creating detection history for the leopard and their prey and livestock detection across all the grids, having 16 replicates each. On each replicate, the detection of the species was coded 1 and nondetection was coded 0. The site covariates were constant in each grid, and we applied *z*‐transformation to normalize the site covariate data. We defined the global model as follows:

$$\text{Global}\left[\left(\hat{\Psi }\right)\left(\text{IO},R,N,\text{PD},\text{PS},W,L\right),\theta_{0}\left(\cdot \right),\theta_{1}\left(\cdot \right),\text{Pt}\left(\text{Samp}\_\text{Eff},\text{IO},R,N,L\right)\right].$$

We identified the suitable covariates on the basis of ecological importance, a recommendation from previous studies, and simplest explanation of model (parsimony). We used a constant model for replicate‐level occupancy parameters (*θ*_0_ and *θ*_1_) (Karanth et al., 2011).

We also could not ignore the possibilities that some of the covariates or other unknown factors influencing the leopard presence contribute to variation in the leopard abundance and hence influence the replicate‐level detectability (Pt). To address this, our occupancy model focused on identifying the suitable covariate model structure for Pt from sample effort (Samp_Eff), management type (IO), ruggedness (*R*), vegetation cover (*N*), and livestock (*L*) using the global model for occupancy. Then, the suitable model structure of Pt was kept constant and $\hat{\Psi }$ was varied for the top covariate model structure on grid‐level occupancy. We modeled covariate stepwise such that if it improved the model fit, then was retained to combine with other covariates in multivariate models that we considered significant from our a priori model building. We applied combination of covariates as additive effects in the model and eliminated models that failed to converge. We identified top competitive models that fit the data well with delta AIC < 2. The competitive models were averaged based on model weights (MacKenzie et al., 2006) to estimate the grid‐specific occupancy, the total fraction of Chure occupied by the leopard, replicate‐level occupancy parameters (“*θ*_0_” and *θ*_1_), and other parameters. We applied the parametric bootstrapping to the untransformed *β* parameter from the top models via simulating 1,000 random deviate to obtain the standard deviation of the mean (MacKenzie et al., 2017, StatDisk 13: Triola Stats, https://www.triolastats.com/).

The distribution of the number of lowland PAs of Nepal is concentrated in Terai Arc Landscape (central, western, and far‐western survey blocks; called “west Chure” hereafter, number of PAs = 5, total area of PAs = 5,331.19 km^2^, *n* = 152 grids in Chure; Figure 1). In the Eastern Block (“east Chure” hereafter, number of PAs = 0, *n* = 71 grids in Chure; Figure 1), a small protected area named Koshi Tappu Wildlife Reserve (KTWR) occurs with a small portion of its northwest boundary touched to Chure range but not included in the survey grids (Figure 1; DNPWC, 2021). These PAs of west Chure bear the leopard source population, and we assumed that the leopard's occupancy is higher compared with the east Chure. Hence, we also separately estimated the leopard occupancy for the east Chure and west Chure. All the covariates described above were used, except the management regime was dropped in the east Chure as no survey grid falls inside the PA., and tiger presence was added in the west Chure. The tigers occupy the protected areas (and some forests outside) of the west Chure (Eisenberg & Lockhart, 1972; Hayward et al., 2006; Pokheral & Wegge, 2019; Ramakrishnan et al., 1999). We followed all the steps and methods as described above (Appendix 8 and Appendix 9 for correlation coefficient “*r*” between covariates of east Chure and west Chure). In the east, there was no spatial correlation in detection, while we checked the performance of the standard occupancy model (MacKenzie et al., 2002) over spatial correlation model (Hines et al., 2010). So, all the analysis was performed using standard occupancy model, whereas in the west Chure, spatial correlation model performed better over standard occupancy model (Table 1) and hence was used for the further analysis.

## 3 RESULTS

The survey team walked a total of 3,244 km to record signs of leopard, their prey, and human disturbances. A combined total of 317 times the signs of leopards were detected in 70 grids from 223 grids surveyed, with a naïve leopard occupancy of 0.31. Wild boars were the most abundant among the prey species with records from 104 grids (48%). They were present in half (49%) of the grids where leopards were detected. Other prey species combined (chital, sambar, rhesus, barking deer) were present in 111 grids (52%). Lopping and encroachment were recorded on 97 grids (45%) whereas livestock sign was detected in 117 grids (55%).

We fit 26 (15 detection and 11 occupancy) a priori alternative model that described expected covariates combination effecting leopard's occupancy and detection. Our result showed the model containing the additive effect of management regime (IO, leopard detection decreased inside the protected areas, opposed our prediction), vegetation cover (NDVI, leopard detection decreased with increase in vegetation cover, as predicted), and livestock presence (*L*, leopard detection increased with increase in livestock presence, opposed our prediction) to be the top detection model (*w* = 0.51; Table 2). The terrain ruggedness and the sampling effort did not influence on the leopard detection. Then, we fixed the top detection model and the role of the covariates on the occupancy ($\hat{\Psi }$) was assessed.

**TABLE 2:** Role of covariates in determining detection probability of leopard sign (Pt) on 2 km long replicates, based on covariates for probability of occurrence of leopard from the global model, $\hat{\Psi }$ (Global) = IO+*R*+*N*+PD+PS+WB+*L*

Model	AIC	ΔAIC	*w*	Model likelihood	*k*
$\hat{\Psi }$ (Global) *θ*_0_(·) *θ*_1_(·) *p* (IO+*N*+*L*) *θ*_0_ *pi* (·)	844.33	0	0.5148	1	30
$\hat{\Psi }$(Global) *θ*_0_(·) *θ*_1_(·) *p*(IO+*N*+*L*+*R*) *θ*_0_ *pi* (·)	846.04	1.71	0.2189	0.4253	31
$\hat{\Psi }$(Global) *θ*_0_(·) *θ*_1_(·) *p*(IO+*N*+*L*+Samp_Eff) *θ*_0_ *pi* (·)	846.19	1.86	0.2031	0.3946	31
$\hat{\Psi }$(Global) *θ*_0_(·) *θ*_1_(·) *p*(IO+*N*) *θ*_0_ *pi* (·)	850.81	6.48	0.0202	0.0392	29
$\hat{\Psi }$ (Global) *θ*_0_(·) *θ*_1_(·) *p*(IO+*L*) *θ*_0_ *pi* (·)	851.35	7.02	0.0154	0.0299	29
$\hat{\Psi }$ (Global) *θ*_0_(·) *θ*_1_(·) *p*(IO+*N*+Samp Eff) *θ*_0_ *pi* (·)	852.63	8.3	0.0081	0.0158	30
$\hat{\Psi }$ (Global) *θ*_0_(·) *θ*_1_(·) *p*(IO+*N*+*R*) *θ*_0_ *pi* (·)	852.71	8.38	0.0078	0.0151	30
$\hat{\Psi }$(Global) *θ*_0_(·) *θ*_1_(·) *p*(IO) *θ*_0_ *pi* (·)	853.75	9.42	0.0046	0.009	28
$\hat{\Psi }$(Global) *θ*_0_(·) *θ*_1_(·) *p*(IO+Samp_Eff) *θ*_0_ *pi* (·)	854.04	9.71	0.004	0.0078	29
$\hat{\Psi }$ (Global) *θ*_0_(·) *θ*_1_(·) *p*(IO+*R*) *θ*_0_ *pi* (·)	854.62	10.29	0.003	0.0058	29
$\hat{\Psi }$(Global) *θ*_0_(·) *θ*_1_(·) *p*(*R*) *θ*_0_ *pi* (·)	887.32	42.99	0	0	28
$\hat{\Psi }$(Global) *θ*_0_(·) *θ*_1_(·) *p*(*L*) *θ*_0_ *pi* (·)	890.43	46.1	0	0	28
$\hat{\Psi }$(Global) *θ*_0_(·) *θ*_1_(·) *p*() *θ*_0_ *pi* (·)	892.94	48.61	0	0	27
$\hat{\Psi }$(Global) *θ*_0_(·) *θ*_1_(·) *p*(Samp_Eff) *θ*_0_ *pi* (·)	894.83	50.5	0	0	28
$\hat{\Psi }$(Global) *θ*_0_(·) *θ*_1_(·) *p*(*N*) *θ*_0_ *pi* (·)	894.93	50.6	0	0	28

Note

$\hat{\Psi }$: model‐averaged leopard occupancy; *p* = replicate‐level detectability; AIC = Akaike's information criterion, ΔAIC = difference in AIC value between the top model and the focal model; *w* = AIC weight; Model likelihood is −2 logarithm of the likelihood function evaluated at maximum; *θ*_0_ = Pr (leopard presence in a replicate/grid occupied and which was absent in the previous replicate) and “*θ*_1_” = Pr (leopard presence in a replicate/grid occupied and was present in the previous replicate); *k* = number of model parameters; Covariates: IO: management regime (grids inside and outside of the protected areas); *R* = terrain ruggedness averaged across each grid; *N* = nondifferent vegetative index averaged across each grid; PD = averaged human population density in each grid; PS = prey species (rhesus, barking deer, chital); WB = wild boar; *L* = livestock presence; Samp_Eff = sampling effort; + = covariates modeled additively; (·) = parameters are held constant.

**FIGURE 2:**
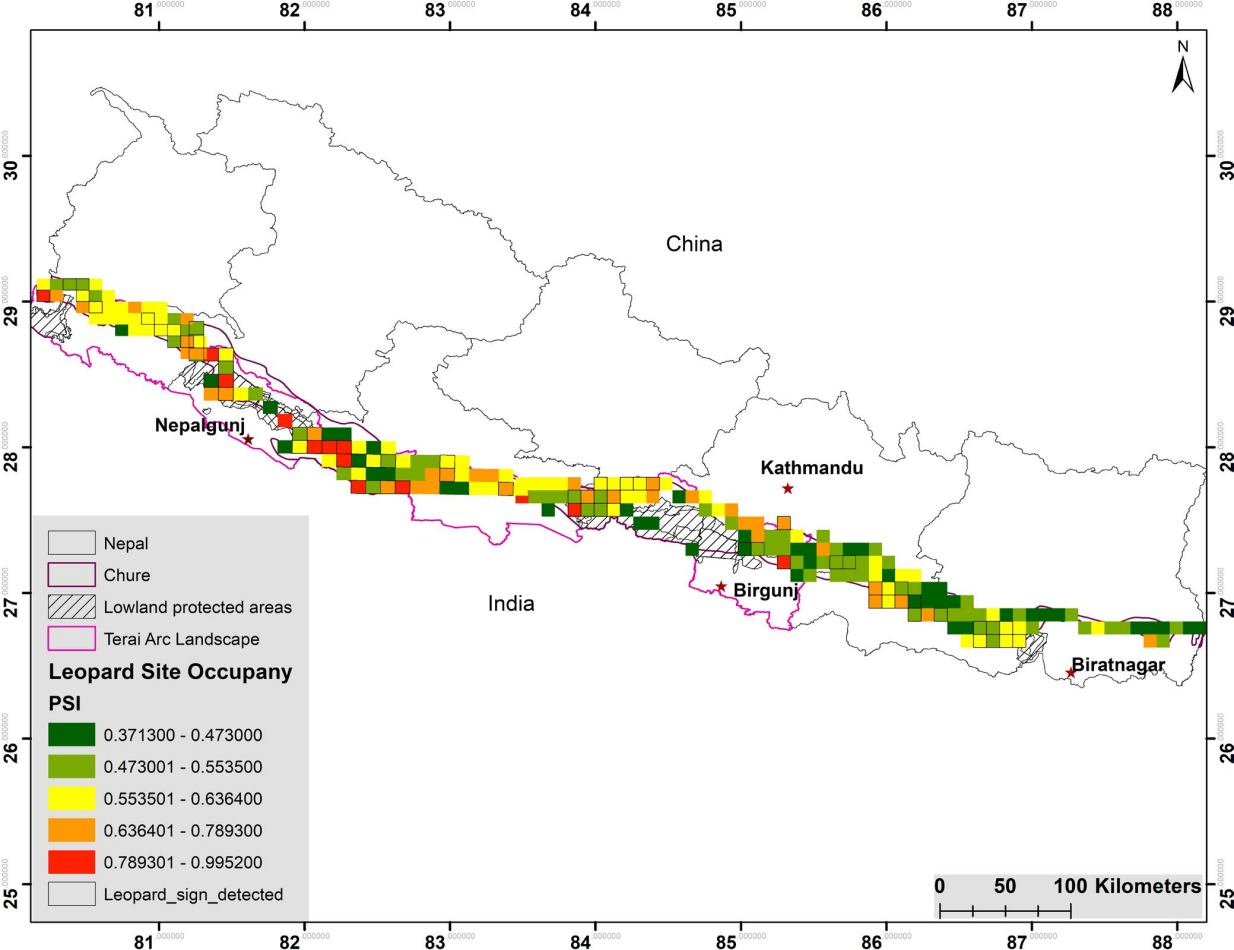
Probability of site occupancy of leopards in the Chure range

The top model for occupancy ($\hat{\Psi }$) of leopard in Chure range obtained after model averaging (*w* = 0.61; Table 3) included wild boar (WB, positive effect, opposed our prediction), human population density (PD, positive effect, opposed our prediction), ruggedness (*R*, positive effect, as predicted), and livestock (*L*, positive effect, opposed our prediction; Table 4). The management regime (IO), vegetation index (NDVI), and prey species (PS) did not influence on the leopard occupancy. The model‐averaged leopard occupancy ($\hat{\Psi }$) in Chure was 0.5732 (*SE* 0.0082) with the detection probability 0.2554 (*SE* 0.1142; Table 5). Thus, the leopard occupied 12,782 km^2^ (*SE* 182 km^2^) potential available habitat of the Chure range. Further, we estimated the grid‐specific occupancy ($\hat{\Psi }$) and variation of leopards across the Chure range (Figure 2).

**TABLE 3:** Model‐specific *β* coefficient estimates for covariates determining leopard detection covariates from the global model, $\hat{\Psi }$ (Global) = IO+*R*+*N*+PD+PS+WB+*L* in the Chure range

Model	*β*‐coefficient estimates for covariates determining leopard detection (*p*) in Chure area Nepal					
	${\hat{\beta }}_{0}$($\hat{\mathrm{SE}}$(${\hat{\beta }}_{0}$))	${\hat{\beta }}_{\text{IO}}$($\hat{\mathrm{SE}}$(${\hat{\beta }}_{\text{IO}}$))	${\hat{\beta }}_{N}$($\hat{\mathrm{SE}}$(${\hat{\beta }}_{N}$))	${\hat{\beta }}_{L}$($\hat{\mathrm{SE}}$(${\hat{\beta }}_{L}$))	${\hat{\beta }}_{R}$($\hat{\mathrm{SE}}$(${\hat{\beta }}_{R}$))	$\hat{\beta }$Samp_Eff($\hat{\mathrm{SE}}$($\hat{\beta }$Samp_Eff))
$\hat{\Psi }$ **(Global) *θ*_0_(·) *θ*_1_(·) *p*(IO+*N*+*L*). *θ*_0_ *pi*(·)**	**−0.962814 (0.796582)**	**−5.200111 (1.596154)**	**−1.298336 (0.434085)**	**1.591561 (0.579693)**	**–**	
$\hat{\Psi }$(Global) *θ*_0_(·) *θ*_1_(·) *p*(IO+*N*+*L*+*R*). *θ*_0_ *pi*(·)	−0.939643 (0.804948)	−5.232734 (1.550219)	−1.442231 (0.538109)	1.712267 (0.639424)	−0.202999 (0.390237)	–
$\hat{\Psi }$(Global) *θ*_0_(·) *θ*_1_(·) p(IO+*N*+*L*+*R*+Samp_Eff). *θ*_0_ *pi*(·)	−0.979584 (0.794018)	−5.261546 (1.584424)	−1.236380 (0.464360)	1.590239 (0.581839)	–	0.120632 (0.321069)

Note

Only the model with ΔAIC < 2 is tabulated. *p* = replicate‐level detectability; *θ*_0_ = Pr (leopard presence in a replicate/grid occupied and which was absent in the previous replicate) and “*θ*_1_” = Pr (leopard presence in a replicate/grid occupied and was present in the previous replicate); Covariates: IO: management regime (grids inside and outside of the protected areas); *R* = terrain ruggedness averaged across each grid; *N* = nondifferent vegetative index averaged across each grid; PD = averaged human population density in each grid; PS = prey species (rhesus, barking deer, chital); WB = wild boar; *L* = livestock presence; *SE* = standard error. + = covariates modeled additively; (·) = parameters are held constant. The *β* coefficients from best model are presented in bold.

**TABLE 4:** Role of covariates in determining probability of leopard occupancy in the Chure range, structured on Pt obtained from Table 2

Model	AIC	**Δ**AIC	*w*	Model Likelihood	*K*
$\hat{\Psi }$(WB+PD+*R*+*L*) *θ*_0_(·) *θ*_1_(·)*p*(IO+*N*+*L*), *θ*_0_ *pi*(·)	858.33	0	0.612	1	27
$\hat{\Psi }$(WB+PD+*R*) *θ*_0_(·) *θ*_1_(·)*p*(IO+*N*+*L*), *θ*_0_ *pi*(·)	859.35	1.02	0.3675	0.6005	26
$\hat{\Psi }$(WB+PD+*L*) *θ*_0_(·) *θ*_1_(·)*p*(IO+*N*+*L*), *θ*_0_ *pi*(·)	866.27	7.94	0.0116	0.0189	26
$\hat{\Psi }$(WB+PD) *θ*_0_(·) *θ*_1_(·)*p*(IO+*N*+*L*), *θ*_0_ *pi*(·)	867.94	9.61	0.005	0.0082	25
$\hat{\Psi }$(WB+*R*) *θ*_0_(·) *θ*_1_(·)*p*(IO+*N*+*L*), *θ*_0_ *pi*(·)	868.46	10.13	0.0039	0.0063	25
$\hat{\Psi }$(WB+*L*) *θ*_0_(·) *θ*_1_(·)*p*(IO+*N*+*L*), *θ*_0_ *pi*(·)	876.36	18.03	0.0001	0.0001	25
$\hat{\Psi }$(WB) *θ*_0_(·) *θ*_1_(·)*p*(IO)(*N*)(*L*) *θ*_0_ *pi*(·)	877.64	19.31	0	0.0001	24
$\hat{\Psi }$(*L*) *θ*_0_(·) *θ*_1_(·)*p*(IO)(*N*)(*L*) *θ*_0_ *pi*(·)	891.68	33.35	0	0	24
$\hat{\Psi }$(PD) *θ*_0_(·) *θ*_1_(·)*p*(IO)(*N*)(*L*) *θ*_0_ *pi*(·)	898.48	40.15	0	0	24
$\hat{\Psi }$(*R*) *θ*_0_(·) *θ*_1_(·)*p*(IO)(*N*)(*L*) *θ*_0_ *pi*(·)	902.44	44.11	0	0	24
$\hat{\Psi }$() *θ*_0_(·) *θ*_1_(·)*p*(IO)(*N*)(*L*) *θ*_0_ *pi*(·)	908.3	49.97	0	0	23

Note

$\hat{\Psi }$: model‐averaged leopard occupancy; *p* = replicate‐level detectability; AIC = Akaike's information criterion, ΔAIC = difference in AIC value between the top model and the focal model; *w* = AIC weight; Model likelihood is −2 logarithm of the likelihood function evaluated at maximum; *θ*_0_= Pr (leopard presence in a replicate/grid occupied and which was absent in the previous replicate) and “*θ*_1_”= Pr (leopard presence in a replicate/grid occupied and was present in the previous replicate); *k* = number of model parameters; Covariates: IO: management regime (grids inside and outside of the protected areas); *R* = terrain ruggedness averaged across each grid; *N* = nondifferent vegetative index averaged across each grid; PD = averaged human population density in each grid; PS = prey species (rhesus, barking deer, chital); WB = wild boar; *L* = livestock presence. In all models, Pt from the top model (Table 2) was modeled as *p*(IO+*N*+*L*); + = covariates modeled additively; (·) = parameters are held constant.

**TABLE 5:** Model‐specific *β* coefficient estimates for covariates determining leopard occupancy in the Chure range

Model	*β*‐coefficient estimates for covariates determining leopard occupancy Ψ in Chure area Nepal				
	${\hat{\beta }}_{0}$($\hat{\mathrm{SE}}$(${\hat{\beta }}_{0}$))	${\hat{\beta }}_{\text{WB}}$ ($\hat{\mathrm{SE}}$(${\hat{\beta }}_{\text{WB}}$))	${\hat{\beta }}_{\text{PD}}$($\hat{\mathrm{SE}}$(${\hat{\beta }}_{\text{PD}}$))	${\hat{\beta }}_{R}$($\hat{\mathrm{SE}}$(${\hat{\beta }}_{R}$))	${\hat{\beta }}_{L}$($\hat{\mathrm{SE}}$(${\hat{\beta }}_{L}$))
$\hat{\Psi }$ **(WB+PD+*R*+*L*) *θ*_0_(·) *θ*_1_(·) *p*(IO+*N*+*L*). *θ*_0_ *pi*(·)**	**−1.067920 (0.599866)**	**2.010155 (0.439844)**	**0.252072 (0.069974)**	**0.264084 (0.095083)**	**0.753054 (0.429026)**
$\hat{\Psi }$(WB+PD+*R*) *θ*_0_(·) *θ*_1_(·) p(IO+*N*+*L*). *θ*_0_ *pi*(·)	−1.073205 (0.601534)	2.226028 (0.413549)	0.240130 (0.069372)	0.260696 (0.094298)	**–**

Note

Only the model with ΔAIC < 2 is tabulated. $\hat{\Psi }$: model‐averaged leopard occupancy; *p* = replicate‐level detectability; *θ*_0_ = Pr (leopard presence in a replicate/grid occupied and which was absent in the previous replicate) and ‘*θ*_1_’ = Pr (leopard presence in a replicate/grid occupied and was present in the previous replicate); Covariates: IO: management regime (grids inside and outside of the protected areas); *R* = terrain ruggedness averaged across each grid; *N* = nondifferent vegetative index averaged across each grid; PD = averaged human population density in each grid; PS = prey species (rhesus, barking deer, chital); WB = wild boar; *L* = livestock presence; *SE* = standard error; + = covariates modeled additively; (·) = parameters are held constant. The *β* coefficients from best model are presented in bold.

In case of east Chure, the top model contained only the ruggedness (*w* = 0.38, *R*, positive, as predicted) as a covariate for detection (*p*_e_) (Appendix 1). In case of west Chure (*p*_w_), the additive effect of management regime (IO, decreased inside the protected areas, opposed our prediction), vegetation index (NDVI, decreased with increased vegetation cover, as predicted), and livestock presence (*L*, leopard detection increased with increased livestock presence, opposed our prediction) to be the top detection model (*w* = 0.53; Appendix 2).

In case of east Chure range, the top model for leopard occupancy (${\hat{\Psi }}_{e}$), obtained after model averaging (*w* = 0.30), was prey species (PS, positive effect on leopard occupancy, as predicted) and vegetation index (NDVI, positive effect, as predicted). The model‐averaged ${\hat{\Psi }}_{e}$ was 0.46 (*SE* 0.043; Appendix 3). Thus, in the east Chure, the leopard occupied the potential available habitat of 3,266 km^2^ (*SE* 311 km^2^) out of 7,100 km^2^ surveyed. Similarly, for the west Chure range, the top model for leopard occupancy (${\hat{\Psi }}_{w}$), obtained after model averaging (*w* = 0.69), was management regime (IO, leopard occupancy increased inside the PAs, as predicted), tiger presence (*T*, leopard occupancy increased in areas with tigers, opposed our prediction), and prey species (PS, leopard occupancy increased with increased prey presence, as predicted). The model‐averaged ${\hat{\Psi }}_{w}$ was 0.70 (*SE* 0.047; Appendix 4). The leopard in the west Chure occupied the potential available habitat of 10,640 km^2^ (*SE* 714 km^2^) out of 15,300 km^2^ surveyed (Table 6).

**TABLE 6:** Estimated occupancy, other parameters, and variance of Chure range

Pr (leopard presence on the 1st replicate), that is, (${\hat{\theta }}_{0}$($\hat{\mathrm{SE}}$(${\hat{\theta }}_{0}$)))	0.2168 (0.0073)
Pr (leopard presence in a replicate/grid occupied and which was absent in the previous replicate), that is, $\hat{\theta }$($\hat{\mathrm{SE}}$($\hat{\theta }$))	0.1292 (0.0073)
Pr (leopard presence in a replicate/grid occupied and was present in the previous replicate), that is, ${\hat{\theta }}_{1}$($\hat{\mathrm{SE}}$(${\hat{\theta }}_{1}$))	0.4726 (0.0086)
Pr (detecting leopard sign on a replicate/grid occupied), that is, $\hat{\mathrm{Pt}}$ ($\hat{\mathrm{SE}}\left(\hat{\mathrm{Pt}}\right)$)	0.2554 (0.1142)
Pr (the total fraction of the area occupied by leopards in the Chure range), that is, $\hat{\Psi }$ ($\hat{\mathrm{SE}}$($\hat{\Psi }$))	0.5732 (0.0082)
Naïve occupancy of leopard from the traditional present/absent approach in the Chure range	0.31

## 4 DISCUSSION

This is the first comprehensive survey of leopard occupancy covering the entire Chure range (~19,000 km^2^) of Nepal. We found the spatial replicate model performed better than the standard occupancy model. Our result showed that more than half of the Chure range was occupied by leopards. Leopard occupancy was higher in the west Chure (0.7) compared with East (0.5). The additive effects of the covariates on the top model influencing the leopard occupancy were the presence of wild boar (one of the prey species, positive—opposed to our assumption), human population density (positive with human density—opposed to our assumption), terrain ruggedness (higher in more rugged area, as we assumed), and the presence of livestock (positive—opposed to our assumption). Similarly, the additive effect of the covariates on the top model influencing the detection probability of the leopard was management regime (higher outside the protected areas—opposed our prediction), vegetation cover (less in the densely vegetated areas—as predicted), and livestock presence (higher in the areas with the presence of livestock—opposed our prediction).

The reliability of the occupancy depends on the detection probability of the sign on the replicates (Hines et al., 2010). The value of naïve estimate occupancy (0.31) through the conventional presence–absence approach created biased in the actual occupancy because it did not consider the false absences (Figure 2). The prior consideration of leopard home range, their behavior, the prior identification of associated covariates while designing the survey, and formation of the representative global model has helped us to obtain robust detection function and explain the pattern of leopard occupancy as well as associated environmental and ecological factors (Karanth et al., 2011).

As assumed, the probability of leopard occurrence (${\hat{\Psi }}_{w}$) in the west Chure range in Terai Arc Landscape (TAL; between Parsa National Park (PNP) in the east and Shuklaphanta National Park (ShNP) was higher ((0.70 (*SE* 0.047))) compared with east Chure range ((0.46 (*SE* 0.043))). There are five national parks with source populations of leopards in west Chure range. Leopards are highly adaptable in terms of foraging strategy and flexible for habitat selection in the rugged Chure area (Balme et al., 2007; Dutta et al., 2013). Similarly, all five national parks are the home for tiger, the apex carnivore, but the leopard occupancy in the west Chure range, inside the protected areas (${\hat{\beta }}_{\text{IO}}$ = 2.62, 0.75 *SE*), showed co‐occurrence with tigers (${\hat{\beta }}_{T}$= 2.93, 1.09 *SE*). Other studies have also documented the high density of leopard co‐occurring with tigers within protected areas through spatial and diet partitioning (Lovari et al., 2015; Odden et al., 2010; Pokheral & Wegge, 2019). Usually, leopards occupy marginal and rugged habitats within protected areas where tiger density is lower (Lamichhane, Persoon, et al., 2019). Further, the rugged terrain of Chure range and the leopard's flexibility to utilize it may reduce their interspecific encounters with tigers (Lamichhane, Leirs, et al., 2019). The prey presence (PS, ${\hat{\beta }}_{\text{PS}}$ = 2.16, 0.70 *SE*) has a positive influence on leopard occupancy of western Chure range. A study on relative abundance of ungulate species (including cattle) in Terai Arc Landscape based on pellet count (pellet groups per 10‐m^2^ plots) documented relatively lower abundance in forest outside the protected areas (PA—2.34 ± 0.15, buffer zone—0.63 ± 0.05, and national forest—0.56 ± 0.03; Shrestha, 2004). Thus, prey could be the determining factor for leopard survival in Chure forests outside the protected areas.

The tiger‐focused conservation activities in protected areas in the west have increased their number nearly twice since 2010 (DNPWC & DFSC, 2018). The increasing number of tigers in these national parks may have pushed leopards to the adjacent Chure range (Lamichhane, Leirs, et al., 2019; Odden et al., 2010; Thapa & Kelly, 2017). A camera trap‐based study in the rugged Chure range within the Chitwan National Park found higher density of leopard (3.3 to 5.1 per 100 km^2^) than tigers (2.3 to 2.9 per 100 km^2^) (Thapa & Kelly, 2017). Besides the TAL area (west Chure range), in the east Chure range of Nepal, there is only a small protected area, that is, Koshi Tappu Wildlife Reserve (area: 349.5 km^2^) which touches a small portion of the Chure range in the northwest (Figure 1). Due to this, the wildlife conservation activities are low in the eastern part. Similarly, the average forest cover in the west Chure range is greater than the east Chure. It may have reduced the prey availability and subsequently reduced the leopard occupancy (${\hat{\Psi }}_{e.}$ = 0.46 (*SE* 0.043)) in the east Chure range compared with the west Chure range (${\hat{\Psi }}_{w}$= 0.70 (*SE* 0.047)). Hence, this study of leopard occupancy distribution helps wildlife managers and policymakers to guide for identifying locations to focus on leopard conservation in the Chure range.

**FIGURE 3:**
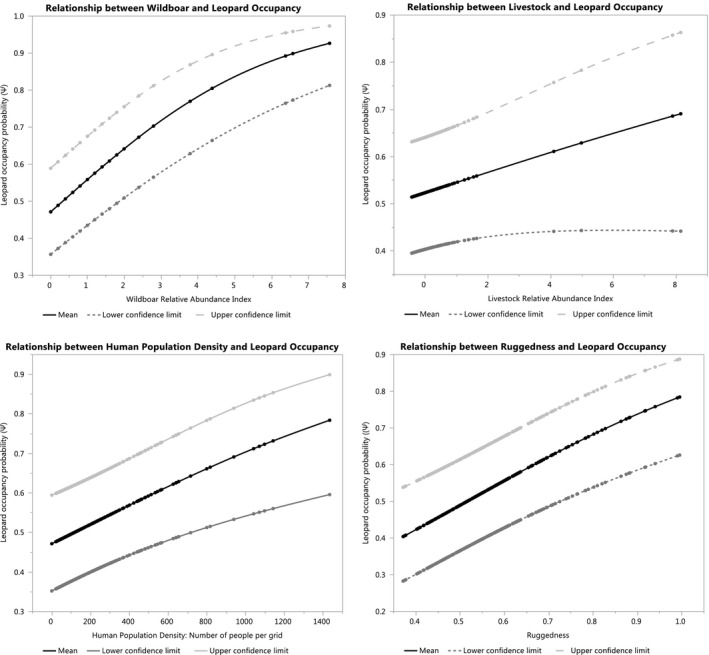
Relationship between top covariates and leopard occupancy ($\hat{\Psi }$) in the Chure range

Our results did not correspond to our a priori hypothesis that leopard avoids wild boar (Eisenberg & Lockhart, 1972; Hayward et al., 2006; Pokheral & Wegge, 2019; Ramakrishnan et al., 1999) but positively influenced the leopard occupancy in the Chure range. The leopard consuming wild boar as a diet was also observed by Kandel et al. (2020) in the Kamdi forest corridor of the western part of the Chure range. The wild boar occurred in almost half of the surveyed grids in the Chure range, the highest among the mammal species surveyed. Leopard and wild boar occurred together in 49 (22%) grids. Our results showed the importance of wild boar as prey species in areas with low prey density for the occurrence of leopard (Figure 3).

We also used other prey species (barking deer, rhesus, and chital) as covariates, but their influence in the model was weak. We believe the rarity of prey other than wild boar in the Chure range is the reason for such results in contrast to our expectation of strong relation between predators (leopard) and prey (Thapa et al., 2021). The opportunistically placed camera traps along with this survey also photographed poachers with guns in various locations. It indicates the widespread hunting of wild prey species (Subedi, Bhattarai, et al., 2021) which have probably contributed to reducing the prey abundance.

The positive influence of the ruggedness index on leopard occupancy of Chure range indicates the extensive use of rugged Chure hills by leopards. The rugged terrain provides an opportunity for ambush predators to hunt (Sharma et al., 2015). Leopards are excellent climbers, and rugged terrain probably does not limit their movements/use of the habitat. Generally flat and less rugged areas are occupied by human settlements, and the rugged hills are still covered with forest providing habitat for leopards, their prey, and other wildlife. However, we did not find the relation between vegetation cover (NDVI) and leopard occupancy. Instead, as our a priori assumption, the detection probability was inversely related to NDVI as the survey was conducted in the postmonsoon season, the time the leaves start shedding from the deciduous trees. These fallen leaves covering the forest floor reduce the chances of detecting the leopard sign in densely vegetated areas. In intact forests (high NDVI value) generally, there are fewer and less visible animal trails. Detecting the leopard sign in such a forest is comparatively difficult which reduces the detection probability. Similarly, the detection of the leopard sign was higher in the Chure range that falls outside the protected areas. It may be because the vegetation cover (NDVI) inside the national park is high in comparison with the outside area, and NDVI has negatively influenced the leopard detection of Chure range (*P*_N_ = −1.29, *SE* 0.43; Krishna et al., 2008).

We found the positive influence of human population density and livestock on leopard occupancy, oppose to our prediction. The majority of the Nepalese rural community is based on agriculture, and livestock is an integral part of their farm (Lamichhane, Persoon, et al., 2019). Livestock was present in ~55% of the surveyed grid and leopard occurred in 19% of the grids with livestock presence. Leopard can persist in highly modified landscape with high human population density (Athreya et al., 2013, 2016; Kuhn, 2014). They adopt different ways to minimize the landscape of fear arose with direct contact with humans in the high human‐disturbed areas (Kerley et al., 2002). Hence, this positive association should not be taken as coexistence but manifestation of high nexus between the animal, habitat, and communities as present in our agrarian society in the landscape, thus prevailing chances of human–wildlife conflict in the Chure range.

Leopards are specialized solitary hunters primarily hunting wild ungulates, but also kill livestock if opportunity arises (Kandel et al., 2020; Treves & Karanth, 2003). In the presence of the sufficient natural prey base, leopards tend to avoid livestock (Kolowski & Holekamp, 2006). We do not have the data on the density of prey in the Chure range but the low detection of prey signs (except the wild boar) indicates their low abundance (Smallwood & Fitzhugh, 1995; Stander, 1998). In the absence of enough wild prey, leopards shift to livestock for diet (Hussain et al., 2019; Khorozyan et al., 2015). Different studies have shown livestock contribution in leopard's diet (Aryal & Kreigenhofer, 2009; Deo, 2014; Harihar et al., 2011; Hussain et al., 2019), and in human‐use landscape, the livestock biomass contribution was even high (Kshettry et al., 2018). Further, the percentage of livestock consumption was high in leopard's diet compared with tigers, where the detection of leopard was positively influenced by livestock (Lamichhane, Leirs, et al., 2019). Also, leopard's detection and occupancy were positively associated with livestock presence in Chitwan National Park, largest lowland PAs of Nepal (Kafley et al., 2019). In our study, the leopard occupancy was positively associated with human population density (${\hat{\Psi }}_{\text{PD}}$ = 0.25, *SE* 0.06), and livestock (L) positively influenced both the leopard occupancy (${\hat{\Psi }}_{L}$ = 0.75, *SE* 0.42) and the detection (*p*_L_ = 1.32, 0.49 *SE*). We suggest that maintaining a sufficient natural prey base can contribute to minimize the livestock depredation and hence decrease the human–leopard conflict in the Chure range.

## 5 CONCLUSION

More than half of the Chure range is occupied by leopards. We identified wild boar, human population density, ruggedness, and livestock presence as top covariates influencing their occupancy that would support the policymakers, researchers, and wildlife managers to search possibilities to increase the leopard occupancy in the range. The grid wise occupancy estimate provides insight to identify the area that needs conservation actions. The positive influence on the occupancy of leopard with the presence of wild boar and livestock has indicated the importance of wild ungulates and pointed the possibilities of human–leopard conflict. The activities focusing to increase the wild prey base in the Chure range through better protection will contribute to reduce the livestock depredation by leopards and threat of their retaliatory killing.

Sign‐based occupancy survey can efficiently assess the spatial distribution of large carnivores such as leopards, providing the direction and effect of covariates governing their presence. Hence, we recommend carrying out the occupancy survey every 5 years across the leopard habitats to understand their status as done for tigers in TAL (Thapa et al., 2021). In future research, the exploration of the livestock depredation and human–leopard conflict data, assessing prey density in leopard habitat via distance sampling or using camera traps (since camera traps capture poachers and also are used to estimate relative prey abundance), and assessing leopard reproductive success and survival/mortality rate inside and outside of the PAs add value to understanding the dynamics of the conflict.

## CONFLICT OF INTEREST

No conflict of interest.

## AUTHOR CONTRIBUTIONS

**Babu Ram Lamichhane:** Conceptualization (equal); Data curation (equal); Formal analysis (equal); Investigation (equal); Methodology (equal); Supervision (equal); Validation (equal); Writing‐review & editing (equal). **Saneer Lamichhane:** Conceptualization (equal); Data curation (equal); Formal analysis (equal); Investigation (equal); Methodology (equal); Writing‐original draft (equal). **Rajan Regmi:** Conceptualization (equal); Writing‐review & editing (equal). **Milan Dhungana:** Conceptualization (equal); Writing‐review & editing (equal). **Shyam Kumar Thapa:** Data curation (equal); Writing‐review & editing (equal). **Anil Prasai:** Data curation (equal). **Aashish Gurung:** Data curation (equal); Writing‐review & editing (equal). **Santosh Bhattarai:** Data curation (equal); Writing‐review & editing (equal). **Rajan Prasad Paudel:** Data curation (equal). **Naresh Subedi:** Conceptualization (equal); Data curation (equal); Formal analysis (equal); Funding acquisition (equal); Investigation (equal); Project administration (equal); Supervision (equal); Validation (equal); Writing‐review & editing (equal).

## Data Availability

Presence/Absence of Leopards in the Chure Range of Nepal. https://doi.org/10.5061/dryad.w0vt4b8s1.

## APPENDIX 1

**Table:** Role of covariates in determining detection probability of leopard sign (Pt) on 2‐km‐long replicates of east Chure

Model	AIC	ΔAIC	*w*	Model Likelihood	*K*
$\hat{\Psi }$(·),*p*(*R*)	249.03	0	0.3824	1	18
$\hat{\Psi }$ (·),*p*(*R*+*N*)	249.99	0.96	0.2366	0.6188	19
$\hat{\Psi }$ (·),*p*(*R*+*L*)	249.99	0.96	0.2366	0.6188	19
$\hat{\Psi }$ (·),*p*(*R*+Samp_Eff)	251	1.97	0.1428	0.3734	19
$\hat{\Psi }$ (·),*p*(·)	262.17	13.14	0.0005	0.0014	17
$\hat{\Psi }$ (·),*p*(*L*)	262.27	13.24	0.0005	0.0013	18
$\hat{\Psi }$ (·),*p*(*N*)	263.75	14.72	0.0002	0.0006	18
$\hat{\Psi }$ (·),*p*(Samp_Eff)	263.96	14.93	0.0002	0.0006	18

Note

$\hat{\Psi }$: model‐averaged leopard occupancy; *p* = replicate‐level detectability; AIC = Akaike's information criterion, ΔAIC = difference in AIC value between the top model and the focal model; *w* = AIC weight; Model likelihood is −2 logarithm of the likelihood function evaluated at maximum; *k* = number of model parameters; Covariates: *R* = terrain ruggedness averaged across each grid; *N* = nondifferent vegetative index averaged across each grid; *L* = livestock presence; Samp_Eff=sampling effort; + = covariates modeled additively; (·) = parameters are held constant. *β*‐coefficient estimates for R from the top detection model = 1.123 (*SE* 0.3).

## APPENDIX 2

**Table:** Role of covariates in determining detection probability of leopard sign (Pt) on 2‐km‐long replicates of west Chure, based on covariates for probability of occurrence of leopard from the global model, $\hat{\Psi }$ (Global) = IO+*R*+*N*+PD+PS+WB+*L*+*T*

Model	AIC	ΔAIC	*w*	Model Likelihood	*K*
$\hat{\Psi }$(Global) *θ*_0_(·) *θ*_1_(·) *p*(IO+*N*+*L*) *θ*_0_ *pi* (·)	614.8	0	0.5317	1	31
$\hat{\Psi }$(Global) *θ*_0_(·) *θ*_1_(·) *p*(IO+*N*+*L*+*R*) *θ*_0_ *pi* (·)	616.68	1.88	0.2077	0.3906	32
$\hat{\Psi }$(Global) *θ*_0_(·) *θ*_1_(·) *p*(IO+*N*+*L*+Samp_Eff) *θ*_0_ *pi* (·)	616.79	1.99	0.1966	0.3697	32
$\hat{\Psi }$(Global) *θ*_0_(·) *θ*_1_(·) *p*(IO+*N*) *θ*_0_ *pi* (·)	621.02	6.22	0.0237	0.0446	30
$\hat{\Psi }$(Global) *θ*_0_(·) *θ*_1_(·) *p*(IO+*N*+*R*) *θ*_0_ *pi* (·)	622.55	7.75	0.011	0.0208	31
$\hat{\Psi }$(Global) *θ*_0_(·) *θ*_1_(·) *p*(IO+*R*) *θ*_0_ *pi* (·)	622.77	7.97	0.0099	0.0186	30
$\hat{\Psi }$(Global) *θ*_0_(·) *θ*_1_(·) *p*(IO+*N*+Samp_Eff) *θ*_0_ *pi* (·)	623.02	8.22	0.0087	0.0164	31
$\hat{\Psi }$(Global) *θ*_0_(·) *θ*_1_(·) *p*(IO+*L*) *θ*_0_ *pi* (·)	623.68	8.88	0.0063	0.0118	30
$\hat{\Psi }$(Global) *θ*_0_(·) *θ*_1_(·) *p*(IO) *θ*_0_ *pi* (·)	625.59	10.79	0.0024	0.0045	29
$\hat{\Psi }$(Global) *θ*_0_(·) *θ*_1_(·) *p*(IO+Samp_Eff) *θ*_0_ *pi* (·)	625.94	11.14	0.002	0.0038	30
$\hat{\Psi }$(Global) *θ*_0_(·) *θ*_1_(·) *p*(*R*) *θ*_0_ *pi* (·)	662.42	47.62	0	0	29
$\hat{\Psi }$(Global), *θ*_0_(·) *θ*_1_(·),*p*(*L*) *θ*_0_ *pi* (·)	668.29	53.49	0	0	29
$\hat{\Psi }$(Global), *θ*_0_(·) *θ*_1_(·),*p*() *θ*_0_ *pi* (·)	670.11	55.31	0	0	28
$\hat{\Psi }$(Global), *θ*_0_(·) *θ*_1_(·),*p*(*N*) *θ*_0_ *pi* (·)	671.97	57.17	0	0	29
$\hat{\Psi }$(Global), *θ*_0_(·) *θ*_1_(·),*p*(Samp_EFF) *θ*_0_ *pi* (·)	672.08	57.28	0	0	29
$\hat{\Psi }$, *θ*_0_(·) *θ*_1_(·),*p*() *θ*_0_ *pi* (·)	676.43	61.63	0	0	20

Note

$\hat{\Psi }$: model‐averaged leopard occupancy; *p* = replicate‐level detectability; AIC = Akaike's information criterion, ΔAIC = difference in AIC value between the top model and the focal model; *w* = AIC weight; Model likelihood is −2 logarithm of the likelihood function evaluated at maximum; *θ*_0_ = Pr (leopard presence in a replicate/grid occupied and which was absent in the previous replicate) and “*θ*_1_” = Pr (leopard presence in a replicate/grid occupied and was present in the previous replicate); *k* = number of model parameters; Covariates: IO: management regime (grids inside and outside of the protected areas); *R* = terrain ruggedness averaged across each grid; *N* = nondifferent vegetative index averaged across each grid; PD = averaged human population density in each grid; PS = prey species (rhesus, barking deer, chital); WB = wild boar; *L* = livestock presence; Samp_Eff = sampling effort; *T* = tiger; + = covariates modeled additively; (·) = parameters are held constant. *β*‐coefficient estimates for IO, *N*, *L* = −4.97 (*SE* 1.34), −1.46(*SE* 0.47), 1.327 (*SE* 0.49), respectively.

## APPENDIX 3

**Table:** Role of covariates in determining probability of leopard occupancy in the east Chure range, structured on Pt obtained from Appendix 1

Model	AIC	ΔAIC	*w*	Model Likelihood	*K*
$\hat{\Psi }$(PS)(*N*),*p*(*R*)	243.64	0	0.3073	1	20
$\hat{\Psi }$(PS),*p*(*R*)	244.27	0.63	0.2242	0.7298	19
$\hat{\Psi }$(PS+*N*+*L*),*p*(*R*)	245.54	1.9	0.1188	0.3867	21
$\hat{\Psi }$(PS+PD),*p*(*R*)	245.86	2.22	0.1013	0.3296	20
$\hat{\Psi }$(PS+*R*),*p*(*R*)	245.96	2.32	0.0963	0.3135	20
$\hat{\Psi }$(PS+*L*),*p*(*R*)	246.13	2.49	0.0885	0.2879	20
$\hat{\Psi }$(·),*p*(*R*)	249.03	5.39	0.0208	0.0675	18
$\hat{\Psi }$(*N*),*p*(*R*)	249.36	5.72	0.0176	0.0573	19
$\hat{\Psi }$(PD),*p*(*R*)	250.64	7	0.0093	0.0302	19
$\hat{\Psi }$(*L*),*p*(*R*)	250.86	7.22	0.0083	0.0271	19
$\hat{\Psi }$(*R*),*p*(*R*)	251.02	7.38	0.0077	0.025	19

Note

$\hat{\Psi }$: model‐averaged leopard occupancy; *p* = replicate‐level detectability; AIC = Akaike's information criterion, ΔAIC = difference in AIC value between the top model and the focal model; *w* = AIC weight; Model likelihood is −2 logarithm of the likelihood function evaluated at maximum; *k* = number of model parameters; Covariates: *R* = terrain ruggedness averaged across each grid; *N* = nondifferent vegetative index averaged across each grid; PD: averaged human population density in each grid; PS: prey species (rhesus, barking deer, chital); WB = wild boar; *L* = livestock presence; In all models, Pt from the top model (Appendix 1) was modeled as *p*(*R*); + = covariates modeled additively; (·) = parameters are held constant. *β*‐coefficient estimates for PS and *N* from the top model determining the leopard occupancy in the east Chure = 3.58(*SE* 1.89) and 0.68 (*SE* 0.48), respectively. The model‐averaged ${\hat{\Psi }}_{e}$ was 0.46 (*SE* 0.043).

## APPENDIX 4

**Table:** Role of covariates in determining probability of leopard occupancy in the west Chure range, structured on Pt obtained from Appendix 2

Model	AIC	ΔAIC	*w*	Model likelihood	*K*
$\hat{\Psi }$(IO+*T*+PS) *θ*_0_(·) *θ*_1_(·) *p*(IO+*N*+*L*) *θ*_0_ *pi*(·)	614.78	0	0.6939	1	26
$\hat{\Psi }$(IO+*T*+PS+*N*), *θ*_0_(·) *θ*_1_(·),*p*(IO+*N*+*L*) *θ*_0_ *pi*(·)	616.58	1.8	0.2821	0.4066	27
$\hat{\Psi }$(IO+*T*), *θ*_0_(·) *θ*_1_(·) *p*(IO+*N*+*L*) *θ*_0_ *pi*(·)	623.99	9.21	0.0069	0.01	25
$\hat{\Psi }$(IO+*T*+*L*) *θ*_0_(·) *θ*_1_(·) *p*(IO+*N*+*L*) *θ*_0_ *pi*(·)	624.2	9.42	0.0062	0.009	26
$\hat{\Psi }$(IO+*T*+PD) *θ*_0_(·) *θ*_1_(·) *p*(IO+*N*+*L*) *θ*_0_ *pi*(·)	624.8	10.02	0.0046	0.0067	26
$\hat{\Psi }$(IO+*T*+*R*) *θ*_0_(·) *θ*_1_(·) *p*(IO+*N*+*L*) *θ*_0_ *pi*(·)	625.98	11.2	0.0026	0.0037	26
$\hat{\Psi }$(IO+*W*) *θ*_0_(·) *θ*_1_(·) *p*(IO+*N*+*L*) *θ*_0_ *pi*(·)	627.07	12.29	0.0015	0.0021	25
$\hat{\Psi }$(IO+*T*+*N*) *θ*_0_(·) *θ*_1_(·) *p*(IO+*N*+*L*) *θ*_0_ *pi*(·)	627.52	12.74	0.0012	0.0017	26
$\hat{\Psi }$(IO+PS), *θ*_0_(·) *θ*_1_(·),*p*(IO+*N*+*L*), *θ*_0_ *pi*(·)	628.11	13.33	0.0009	0.0013	25
$\hat{\Psi }$(IO), *θ*_0_(·) *θ*_1_(·),*p*(IO+*N*+*L*), *θ*_0_ *pi*(·)	640.82	26.04	0	0	24
$\hat{\Psi }$(IO+*R*), *θ*_0_(·) *θ*_1_(·),*p*(IO+*N*+*L*), *θ*_0_ *pi*(·)	641.08	26.3	0	0	25
$\hat{\Psi }$(PS), *θ*_0_(·) *θ*_1_(·),*p*(IO+*N*+*L*), *θ*_0_ *pi*(·)	641.23	26.45	0	0	24
$\hat{\Psi }$(IO+*N*), *θ*_0_(·) *θ*_1_(·),*p*(IO+*N*+*L*), *θ*_0_ *pi*(·)	642.43	27.65	0	0	25
$\hat{\Psi }$(IO+PD), *θ*_0_(·) *θ*_1_(·),*p*(IO+*N*+*L*), *θ*_0_ *pi*(·)	642.77	27.99	0	0	25
$\hat{\Psi }$(IO+*L*), *θ*_0_(·) *θ*_1_(·),*p*(IO+*N*+*L*), *θ*_0_ *pi*(·)	647.88	33.1	0	0	25
$\hat{\Psi }$(*W*), *θ*_0_(·) *θ*_1_(·),*p*(IO+*N*+*L*), *θ*_0_ *pi*(·)	648.16	33.38	0	0	24
$\hat{\Psi }$(*L*), *θ*_0_(·) *θ*_1_(·),*p*(IO+*N*+*L*), *θ*_0_ *pi*(·)	648.2	33.42	0	0	24
$\hat{\Psi }$(*T*), *θ*_0_(·) *θ*_1_(·),*p*(IO+*N*+*L*), *θ*_0_ *pi*(·)	648.76	33.98	0	0	24
$\hat{\Psi }$, *θ*_0_(·) *θ*_1_(·),*p*(IO+*N*+*L*), *θ*_0_ *pi*(·)	650.37	35.59	0	0	23
$\hat{\Psi }$(*N*), *θ*_0_(·) *θ*_1_(·),*p*(IO+*N*+*L*), *θ*_0_ *pi*(·)	650.67	35.89	0	0	24
$\hat{\Psi }$(*R*), *θ*_0_(·) *θ*_1_(·),*p*(IO+*N*+*L*), *θ*_0_ *pi*(·)	650.79	36.01	0	0	24
$\hat{\Psi }$(PD), *θ*_0_(·) *θ*_1_(·),*p*(IO+*N*+*L*), *θ*_0_ *pi*(·)	651.02	36.24	0	0	24

Note

$\hat{\Psi }$: model‐averaged leopard occupancy; *p* = replicate‐level detectability; AIC = Akaike's information criterion, ΔAIC = difference in AIC value between the top model and the focal model; *w* = AIC weight; Model likelihood is −2 logarithm of the likelihood function evaluated at maximum; *θ*_0_ = Pr (leopard presence in a replicate/grid occupied and which was absent in the previous replicate) and “*θ*_1_”= Pr (leopard presence in a replicate/grid occupied and was present in the previous replicate); *k* = number of model parameters; Covariates: IO: management regime (grids inside and outside of the protected areas); *R* = terrain ruggedness averaged across each grid; *N* = nondifferent vegetative index averaged across each grid; PD: averaged human population density in each grid; PS: prey species (rhesus, barking deer, chital); WB = wild boar; *L* = livestock presence; *T* = tiger; In all models, Pt from the top model (Appendix 2) was modeled as *p*(IO+*N*+*L*); + = covariates modeled additively; (·) = parameters are held constant. Model‐specific *β*‐coefficient estimates for covariates IO, *T*, PS from the top model determining leopard occupancy in the west Chure = 2.62 (*SE* 0.75), 2.93 (*SE* 1.09), and 2.16 (*SE* 0.70), respectively.

## APPENDIX 5

**Table:** Estimated occupancy, other parameters, and variance in the west Chure

Pr (leopard presence in the 1st replicate), that is, (${\hat{\theta }}_{0}$($\hat{\mathrm{SE}}$(${\hat{\theta }}_{0}$)))	0.1711 (0.027)
Pr (leopard presence in a replicate/grid occupied and which was absent in the previous replicate), that is, $\hat{\theta }$($\hat{\mathrm{SE}}$($\hat{\theta }$))	0.1404 (0.091)
Pr (leopard presence in a replicate/grid occupied and was present in the previous replicate), that is, ${\hat{\theta }}_{1}$($\hat{\mathrm{SE}}$(${\hat{\theta }}_{1}$))	0.615 (0.009)
Pr (detecting leopard sign on a replicate/grid occupied), that is, $\hat{\mathrm{Pt}}$($\hat{\mathrm{SE}}$($\hat{\mathrm{Pt}}$))	0.3170 (0.299)
Pr (the total fraction of the area occupied by leopards in the west Chure range), that is, $\hat{\Psi }$ ($\hat{\mathrm{SE}}$($\hat{\Psi }$))	0.7069 (0.047)
Naïve occupancy of leopard from the traditional present/absent approach in the west Chure range	0.34

## APPENDIX 6

**Table:** Definition and predicted effect of covariates in detectability and occupancy of leopard of Chure range

Covariates	Definition	Type	Expected effect	Remarks
Prey abundance	The relative abundance of prey species (barking deer, chital, wild boar, rhesus) across the 2‐km continuous random transect	Continuous	Positive (Ψ, known to be the function of carnivore densities, Karanth et al., 2011)	In case of wild boar, we separated it from rest prey as it is often avoided by predators for its aggressive behavior, so we expected negative effect on Ψ
Tiger	The relative abundance of tiger in the west Chure range	Continuous	Negative for Ψ, as leopard avoids tiger	The tigers are present only in the west Chure range
Human disturbance	Lopping and encroachment of humans across entire grid	Continuous	Negative(Ψ, Predators avoid human disturbance, Muhly et al., 2011)	
Livestock presence	The relative abundance of livestock (cow, goat) across the 2‐km continuous random transect	Continuous	Negative for both Ψ and “*p*” (overgrazing by livestock is threat to prey species; considered as substitute for human impacts, Karanth et al., 2011)	
Management Regime	The location of survey grids inside or outside of the protected areas of the Chure range (inside = 1, outside = 0)	Categorical	Positive for both Ψ and *p* (the human disturbance inside the protected areas are low compared to nonprotected areas)	
Normalized difference Vegetation Index (NDVI)	Averaged across grid cell. Calculated from 250‐m resolution Medium Resolution Imaging Spectroradiometer (MODIS) satellite images	Continuous	Positive for Ψ (provides an opportunity for leopard, ambush predator) to hunt (Sharma et al., 2015), negative “*p*” (our study period was postmonsoon, and the leaves shading from the deciduous tree during this season reduces the chances of sign detection)	Arc GIS 10.1
Terrain Ruggedness (*R*)	Averaged across grid cell. Calculated using 90 m ASTER DEM.	Continuous	Positive for Ψ and “*p*” (increased ruggedness will be harder for people to access and disturbance will be lower)	Arc GIS 10.1
Human Population Density (PD)	Averaged across grid cell. Calculated from the Gridded Population of the World Version 4	Continuous	Negative for Ψ (increased population density within a grid increases the human disturbance)	Arc GIS 10.1
Sampling Effort (SE)	Total km of continuous random transect walk actually surveyed in a grid	Continuous	Positive for “*p*” (survey effort varied from grid to grid due to logistical constraint (Harihar & Pandav, 2012)	

## APPENDIX 7

**Table:** Correlation coefficient (*r*) value between the covariates in the Chure range. It was calculated using PAST version (4.0). When a set of two covariates have |*r*| ≥ .7, one was dropped from the analysis. The correlation coefficient between human disturbance (HD) and livestock presence (*L*) was >0.7 (bold), so HD was dropped off. Other covariates: Samp_Eff=sample effort, IO = management regime (inside and outside of PA), *R* = ruggedness, PD = human population density, Leo = leopard, PS = prey species, WB = wild boar

	Samp_Eff	IO	*R*	*N*	PD	Leopard	PS	WB	*L*
Samp_Eff									
IO	−0.14								
*R*	0.21	−0.15							
*N*	0.06	0.11	−0.20						
PD	−0.05	−0.01	−0.12	−0.11					
Leopard	0.24	0.18	0.09	0.12	0.16				
PS	0.32	0.22	−0.02	0.05	0.11	0.41			
WB	0.29	0.21	−0.06	0.25	0.04	0.38	0.45		
*L*	0.41	−0.08	0.25	0.29	−0.07	0.20	0.16	0.26	
HD	0.35	−0.05	0.24	0.31	−0.11	0.14	0.19	0.12	**0.84**

## APPENDIX 8

**Table:** Correlation coefficient (*r*) value between the covariates in the east Chure range. It was calculated using PAST version (4.0). When a set of two covariates have |*r*| ≥ .7, one was dropped from the analysis. The correlation coefficient between human disturbance (HD) and livestock presence (*L*) was >0.7 (bold), so HD was dropped off. Other covariates: Samp_Eff = sample effort, *R* = ruggedness, *N* = NDVI, PD = human population density, Leo = leopard, PS = prey species, WB = wild boar

	Samp_Eff	*R*	*N*	PD	Leo	PS	WB	*L*
Samp_Eff								
*R*	0.05							
NDVI	−0.07	−0.30						
PD	0.04	−0.56	−0.01					
Leo	0.23	0.29	−0.01	−0.15				
PS	0.34	0.10	−0.06	0.05	0.56			
WB	0.29	0.14	−0.13	0.17	0.41	0.46		
*L*	0.32	0.21	−0.10	−0.29	0.43	0.40	0.20	
HD	0.29	0.19	−0.04	−0.27	0.51	0.38	0.19	**0.91**

## APPENDIX 9

**Table:** Correlation coefficient (*r*) value between the covariates in the west Chure range. It was calculated using PAST version (4.0). When a set of two covariates have |*r*| ≥ .7, one was dropped from the analysis. The correlation coefficient between human disturbance (HD) and livestock presence (*L*) was >0.7 (bold), so HD was dropped off. Other covariates: Samp_Eff = sample effort, IO = management regime (inside and outside of PA), *R* = ruggedness, *N* = NDVI, PD = human population density, Leo = leopard, PS = prey species, WB = wild boar, *T* = tiger

	Samp_Eff	IO	*R*	*N*	PD	Leo	PS	WB	*L*	HD
Samp_Eff										
IO	−0.15									
*R*	0.26	−0.22								
*N*	0.16	0.05	−0.23							
PD	−0.24	−0.02	−0.44	−0.03						
Leo	0.25	0.19	−0.02	0.15	−0.05					
PS	0.32	0.28	−0.06	0.12	0.01	0.36				
WB	0.35	0.20	−0.22	0.27	−0.04	0.37	0.54			
*L*	0.46	−0.11	0.21	0.43	−0.22	0.12	0.09	0.25		
HD	0.37	−0.05	0.25	0.48	−0.29	0.01	0.12	0.13	**0.83**	
*T*	0.04	0.22	−0.21	0.04	0.11	0.35	0.38	0.26	−0.21	−0.23
